# The Decoding Toolbox (TDT): a versatile software package for multivariate analyses of functional imaging data

**DOI:** 10.3389/fninf.2014.00088

**Published:** 2015-01-06

**Authors:** Martin N. Hebart, Kai Görgen, John-Dylan Haynes

**Affiliations:** ^1^Department of Systems Neuroscience, University Medical Center Hamburg-EppendorfHamburg, Germany; ^2^Bernstein Center for Computational Neuroscience, Charité UniversitätsmedizinBerlin, Germany; ^3^Berlin Center for Advanced Neuroimaging, Charité UniversitätsmedizinBerlin, Germany; ^4^Berlin School of Mind and Brain, Humboldt-Universität zu BerlinBerlin, Germany; ^5^Fachgebiet Neurotechnologie, Technische Universität BerlinBerlin, Germany

**Keywords:** multivariate pattern analysis, decoding, pattern classification, fMRI, representational similarity analysis, searchlight

## Abstract

The multivariate analysis of brain signals has recently sparked a great amount of interest, yet accessible and versatile tools to carry out decoding analyses are scarce. Here we introduce *The Decoding Toolbox* (TDT) which represents a user-friendly, powerful and flexible package for multivariate analysis of functional brain imaging data. TDT is written in Matlab and equipped with an interface to the widely used brain data analysis package SPM. The toolbox allows running fast whole-brain analyses, region-of-interest analyses and searchlight analyses, using machine learning classifiers, pattern correlation analysis, or representational similarity analysis. It offers automatic creation and visualization of diverse cross-validation schemes, feature scaling, nested parameter selection, a variety of feature selection methods, multiclass capabilities, and pattern reconstruction from classifier weights. While basic users can implement a generic analysis in one line of code, advanced users can extend the toolbox to their needs or exploit the structure to combine it with external high-performance classification toolboxes. The toolbox comes with an example data set which can be used to try out the various analysis methods. Taken together, TDT offers a promising option for researchers who want to employ multivariate analyses of brain activity patterns.

## Introduction

Human neuroscientists are interested in understanding the function of the human brain and nervous system. For that purpose, they have developed numerous methods that directly or indirectly measure the activity of the nervous system at work. One of these methods is functional magnetic resonance imaging (fMRI) which measures brain activity indirectly through the blood oxygen level dependent (BOLD) response (Logothetis et al., 2001). Conventionally, the focus of fMRI has been to perform mass-*univariate* analyses, i.e., to analyze the recorded data time courses of each fMRI brain voxel (for MEG/EEG each sensor/electrode) separately, for example with the general linear model (GLM, e.g., Friston et al., 1994).

More recently, these mass-*univariate* analysis methods have been complemented by *multivariate pattern analysis* (MVPA) which refers to a collection of multivariate methods of brain data analysis that incorporate multiple dependent variables at the same time (Haynes and Rees, 2006; Norman et al., 2006; Kriegeskorte, 2011). One of the most popular multivariate methods of brain data analysis is generally referred to as multivariate *decoding* which describes the mapping of multiple dependent variables to one or multiple independent variables, and which contrasts with multivariate *encoding* describing the opposite mapping (Naselaris et al., 2011). The popularity of multivariate methods of brain data analysis stems from three facts: First, multivariate methods offer increased *sensitivity* in detecting statistical dependence between cognitive variables and (patterns of) brain activity, because these methods can combine information across multiple voxels and exploit their covariance. Put simply, multivariate methods make it easier to detect existing differences between brain signals. Second, multivariate methods allow for greater *specificity* in finding a statistical dependence between measured brain data and multiple categorical responses. Although a number of brain regions show overall changes in activity selective for specific stimulus categories (e.g., Kanwisher et al., 1997), stimuli can be encoded in a more distributed manner (Averbeck et al., 2006). Multivariate methods can be used to target distributed representations of stimuli, cognitive variables, or other variables of interest. This effectively enables researchers to ask more specific questions regarding neuronal representations, for example about the representation of individual face exemplars (Kriegeskorte et al., 2007; Nestor et al., 2011). Third, multivariate decoding methods allow functional neuroimaging to be used for accurate prediction of cognitive and mental states in the form of a “neuronal marker.” For example, they might be used to support the diagnosis of neurological disorders beyond regions typically detected by radiologists (Weygandt et al., 2011), predict the conversion from mild cognitive impairment to Alzheimer's disease (Cui et al., 2011), interrogate cognitive states from people that cannot communicate (Horikawa et al., 2013), or support non-invasive brain-computer interfaces for motor control in paralyzed patients (Blankertz et al., 2007).

Although the number of publications using multivariate decoding methods has been rising continuously, it still seems to be common for researchers to program their own multivariate data analysis pipeline from scratch^1^. This is not only very time-consuming and redundant, it is also very error-prone. Indeed, if the same piece of code is adapted for different decoding analyses, changes that were done for previous analyses might be overlooked in later analyses or might affect other analysis steps which can produce invalid results. Just as problematic—and not uncommon even for experts—is the unintentional wrong use of decoding methods, for example circular analyses (Kriegeskorte et al., 2009). In addition, researchers might hesitate to conduct decoding analyses, either because of their limited understanding of machine learning methods for functional neuroimaging (covered in detail by Formisano et al., 2008; Pereira et al., 2009; Lemm et al., 2011) or because of the effort involved in learning to apply a new analysis method. For that reason, researchers getting started with decoding would strongly benefit from an easy-to-use implementation of multivariate methods. At the same time, they would ideally keep using the same software for simple analyses that they would later use for more sophisticated approaches once they have gained sufficient knowledge.

Here we present *The Decoding Toolbox* (TDT) that offers a flexible yet easy to use framework for decoding analyses in Matlab (Mathworks, Natick, MA). TDT offers an interface with SPM (Statistical Parametric Mapping, http://www.fil.ion.ucl.ac.uk/spm/), the most common fMRI data analysis software, but can also be extended to other fMRI data analysis packages. TDT can be used by any researcher with basic knowledge in fMRI data analysis and minimal programming skills, while at the same time allowing a large amount of flexibility in the application of complex decoding approaches for more experienced researchers. We briefly summarize the key features of the toolbox that should make its use attractive.

### Simplicity

Originally, we created TDT to simplify setting up decoding analyses and prevent the unintentional introduction of programming errors or unnoticed changes in settings that do not elicit error messages, but which may severely compromise results. For that reason, we wanted to make TDT particularly easy to use, where all important settings are made at the beginning. We believe that minimal programming ability is necessary, with the only requirement being that a user is able to modify existing Matlab scripts that define the settings for the decoding analysis. This simplicity also allows users to see at a glance what decoding analysis they are performing.

### Modular structure and transparency

The modular structure and use of encapsulation for different sub-routines make the toolbox relatively easy to understand. At the same time, this structure is less error prone and reduces programming effort from the side of developers. For users, the existence of decoding design matrices (see below) and the possibility of extensive logging make the internal processes of the toolbox more transparent, which also reduces the probability of mistakes.

### SPM interface

TDT has been developed from an SPM perspective and works with current SPM versions (downwards compatible to SPM2), but can also be used outside of SPM. TDT can directly read all decoding-relevant information from SPM design matrices and in that way simplifies the process of setting up standard decoding analyses. For example, a complete leave-one-run-out cross-validation decoding analysis can be executed using one line of code, by providing the path to the analysis and the names of the regressors that should be classified (see below).

### Modularity, versatility, extendibility

**Figure 1:**
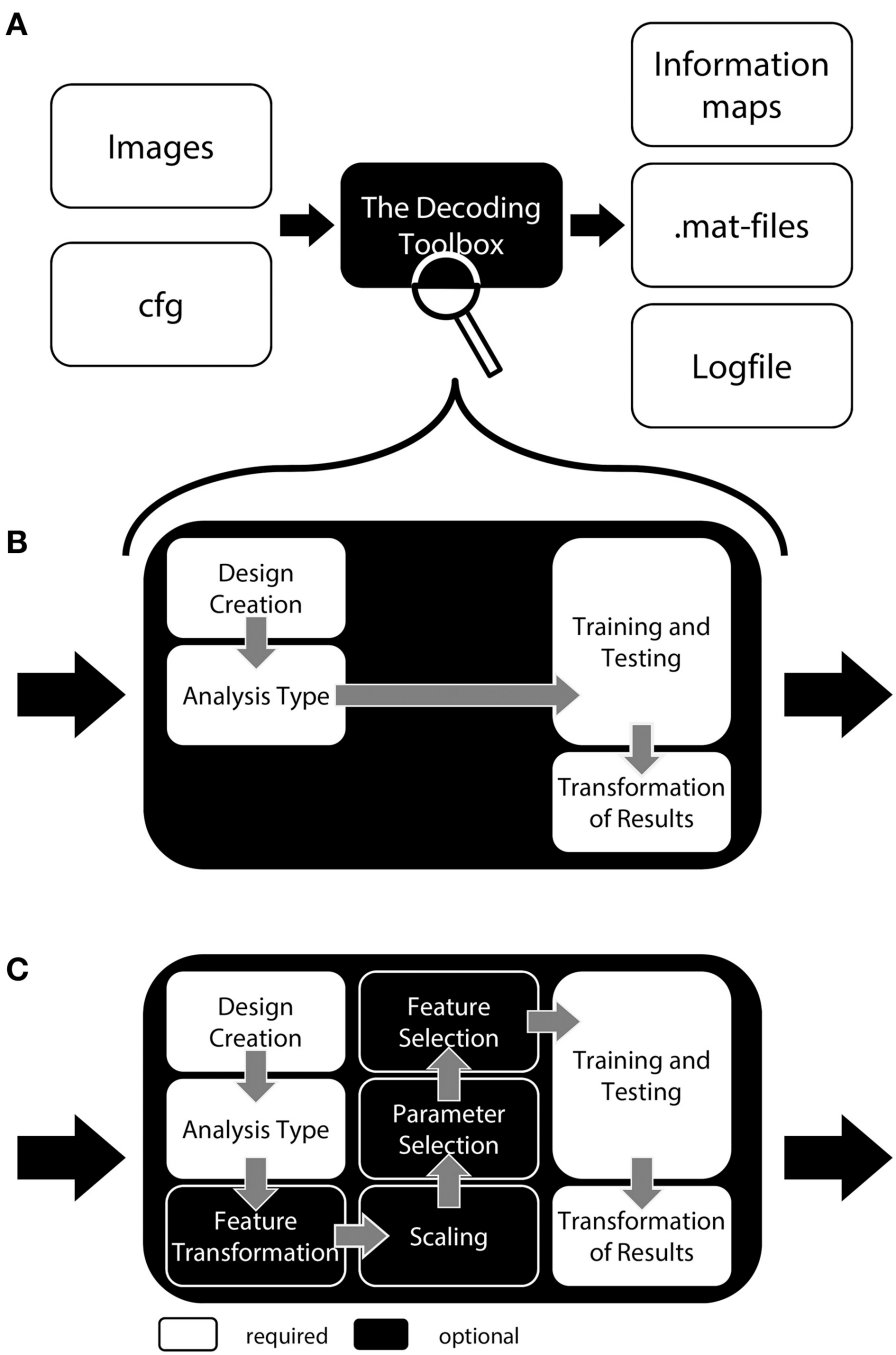
**General structure of The Decoding Toolbox (TDT). (A)** TDT in the view of basic users. All that is required are brain images (ideally preprocessed with SPM) and a configuration variable *cfg* that contains all decoding-relevant information. TDT will then generate results, including. mat-files with the results or if required brain maps displaying the decoded information in space. **(B)** TDT view for intermediate users. Decoding design creation, type of analysis, type of classifier and type of output can be modified. All of these settings are necessary for any decoding analysis, which will be set to default settings if not specified by the user. This level of description already covers most scenarios that the typical user would encounter. **(C)** All TDT options. For the optional functions including feature selection, feature transformation, scaling, and parameter selection, TDT offers a number of preconfigured settings which can be customized. Expert users can extend the toolbox to include new methods (e.g., classifiers, feature selection methods) or can even create an interface to external machine learning packages.

TDT comes with a number of methods which allow for executing most commonly used analysis pipelines “out of the box” (Figure 1). These include feature scaling, feature transformation (e.g., principal component analysis), parameter selection, and feature selection as steps prior to decoding. The decoding analysis itself has two-class and multiclass classification implemented, using a number of often-used classifiers including support vector classifiers, logistic regression, and correlation classifiers (Haxby et al., 2001). Additionally, support vector regression is part of the toolbox. The toolbox has been tested and runs under Windows, Linux, and Mac OS.

Due to the modular structure and the simplicity of the code, users can extend TDT with little effort to incorporate other methods as well: Other software packages can be used instead of SPM, new classifiers can be introduced, other means of feature selection can be applied, and even complete toolboxes—e.g., other Matlab decoding toolboxes—can be interfaced to run in the TDT framework.

We chose to implement our toolbox in Matlab because it is a very popular high-level programming language in the neuroimaging community and offers an excellent programming environment with a large and helpful community. We are aware that part of the neuroimaging community is trying to move toward completely non-commercial software instead. TDT does, however, not require any additional commercial Matlab toolboxes such as the Statistics or Bioinformatics Toolbox. Many functions were written specifically for TDT, for example calculation of *F*-values, implementation of statistical distributions, and quick 1-D linear interpolation.

### Speed

TDT has been profiled extensively to identify and remove possible bottlenecks that would otherwise reduce the speed of decoding analyses. For example, TDT can achieve a three-fold speed increase in cross-validation designs by computing a kernel (Müller et al., 2001) only once and then passing the training and test part of that kernel to the classifier. The resulting speed improvement becomes particularly apparent with large classification problems or searchlight analyses. In addition, we rewrote many built-in Matlab functions, for example for creating correlation matrices or finding unique values, considerably speeding-up processing.

### Strong debugging and error checking

We wanted to make sure that there are as few programming errors as possible in the code, a goal that is particularly difficult given the flexibility and diversity of possible decoding analyses. Although in general it is never possible to guarantee that any software is free from bugs, we regularly run a number of basic analyses as benchmarks to check if these analyses still run and create the same output as in previous analyses. TDT has been extensively tested by many beta users and, importantly, several published results used prior versions of the toolbox (Hesselmann et al., 2011; Christophel et al., 2012, in press; Hebart et al., 2012, in press; Reverberi et al., 2012; Christophel and Haynes, 2014; Ritter et al., 2014; Van Kemenade et al., 2014a,b; Ludwig et al., 2015; Bilalić et al., in press; Guggenmos et al., in press).

In addition, TDT contains many checks to prevent erroneous analyses. In general, it is always possible that unwanted errors occur, and of course researchers always have to double-check if their analysis yields meaningful results. The implemented checks facilitate the process of detecting and eliminating errors. Among others, they ensure independence of training and test data, that all data has the same realignment parameters, and more generally whether the selected options are incomplete or would generate errors. Other initial checks help in saving time and frustration of the user: Each decoding analysis creates a log-file which can be used to recover possible sources of errors. These features have been implemented based on the experience of the authors as well as through user experience and will continuously be improved.

### Readability

TDT is open-source (see License Statement), with extensively commented code, comprehensive variable names, and an execution structure that follows along a main function with several auxiliary functions. We did not want to provide a black-box where a user cannot follow the steps that are carried out in a decoding analysis.

In the following we will describe TDT in more detail. We will use the style of a tutorial which is intended to ease readability of this section. We will introduce TDT at three different levels of complexity, starting with a rough description of the toolbox to provide an initial overview of the basic structure of TDT. This section should be sufficient for users just getting started with decoding, or those who would like to conduct a simple generic decoding analysis. Second, we give a more detailed explanation of how to expand on this standard decoding analysis for users at an intermediate level who want to tailor the analysis to their needs. Third, for advanced users we expand on more detailed optional settings of the toolbox, including parameter selection or feature selection. After these three steps, we will briefly demonstrate how users who want to exploit the full capabilities of the toolbox can extend the toolbox to new classifiers, new feature selection methods, or even complete machine learning packages.

We may use terminology unfamiliar to some readers. Rather than explaining all terms in the text, we summarize the most important ones in Table 1 (for a detailed explanation of classification-related terms, please consult Pereira et al., 2009). Although we provide examples written for users who want to carry out within-subject classification, most of these examples also hold for between-subject classification.

**Table 1:** **Important terminology for multivariate decoding with The Decoding Toolbox**.

**Term**	**Description**
Beta maps	After estimating the *general linear model*, each regressor in each voxel receives a parameter estimate *beta* which can be used for classification and reflects the fit of the model to the data in form of a regression coefficient. Since each voxel is analyzed separately, brain images of betas (i.e., *beta maps*) are created for each regressor
Chunk	A unit that determines which data should remain together within a *decoding step* (often: *cross-validation* fold). Typically, *chunks* are used to assign run numbers to data, which in *cross-validation* should ensure that data is independent and not biased by temporal autocorrelation. For a typical *leave-one-run-out cross-validation*, there are as many *chunks* as there are *decoding steps*. For methods where data samples are drawn repeatedly, the number of *decoding steps* is often a lot larger than the number of *chunks*
Cross-classification	Not to be confused with *cross-validation*. A method to test the stability of a classifier, which can be used (1) to demonstrate the generality of a classifier, (2) for a representational association between two cognitive functions, or (3) simply to show representational stability across time
Cross-validation	Method to estimate of how well a classifier generalizes to novel data. In *leave-one-run-out cross-validation*, data is partitioned in n_runs_ *chunks*, and on each *decoding step* (also called *fold* or iteration) one *chunk* is used as test data to evaluate a classifier trained on all other chunks. Typically, the average performance of all *decoding steps* in terms of classification accuracy is then used to evaluate the classifier. *Nested cross-validation* is cross-validation that is done only on training data to optimize the performance of the classifier (e.g., in *feature* or *parameter selection*)
Curse of dimensionality	Within machine learning the fact that classifier performance, i.e., the predictive power of a classifier drops when the number of features (e.g., voxels) becomes much larger than the number of samples (e.g., brain images)
Decoding step	An iteration of a decoding analysis which is part of the cycle of evaluating the classifier. When *cross-validation* is used, *decoding step* refers to a cross-validation fold
Feature selection	Methods that reduce the number of features (e.g., voxels). In our terminology, selecting *regions of interest* or running a *searchlight analysis* are not parts of *feature selection*. In addition, we treat methods separately that reduce the dimensionality, but give up the voxel-to-voxel mapping (e.g., PCA). Within TDT, we refer to methods that change the voxel-to-voxel mapping as *feature transformation* methods
General linear model (GLM)	A statistical model that incorporates analysis of variance, linear regression, and related parametric tests into a common framework. In brain imaging, the GLM is commonly used to explain each voxel's time series separately with multiple linear regressors each representing conditions of interest or nuisance variables (Friston et al., 1994). The term *mass-univariate* refers to the fact that the GLM is calculated for each voxel individually
Hyperplane	A plane in more than 3D. Typically, the term *separating hyperplane* is used which separates the space spanned by different features (e.g., voxels) in two subspaces and defines the decision boundary of the classifier. Each part of the voxel space is in this way assigned to one of two classes
*L*1/*L*2-norm	A regularization method which influences the complexity of a classification model. In most cases, an *L*2-norm is used which minimizes length of the *weight vector*. The *L*1-norm can be used to minimize the sum of the absolute weights, typically resulting in a sparser model (i.e., less features will contribute to the classification)
Margin	For *support vector machines*, the area of space between two classes, of which the center is typically the separating hyperplane. SVMs have the goal of maximizing the margin between two classes
Searchlight analysis	One of the three most common types of decoding analysis conducted, the two other being *whole brain decoding* and *region-of-interest decoding*. *Searchlight decoding* typically creates a map of classification accuracies that can be interpreted as the local information content around each voxel (Kriegeskorte et al., 2006; Haynes et al., 2007)
Support vector machine	A type of classifier that maximizes the *margin* between two different classes (Cortes and Vapnik, 1995)
Weight vector	Determines the contribution of each feature to the final classifier function. For most classifiers, the weight vector cannot be directly interpreted as reflecting the classified variable, because it is a filter that extracts a class signal while at the same time it suppresses correlated noise. Using the covariance of the data, the weight vector can be converted into an interpretable *pattern vector* (Haufe et al., 2014) which in case of voxel features can be mapped back to a brain image

## A simple decoding analysis

For a general decoding analysis, let us for the moment treat TDT as a black box (Figure 1A). The user feeds in a set of brain images that he wants to classify and receives a single classification accuracy or an information map as output. What is needed in addition is the configuration variable *cfg* which carries all information necessary to conduct a specific decoding analysis. Most parameters in *cfg* are defaults that are set automatically; parameters are only set manually when they should be changed. In addition, the SPM-interface can automatically extract the decoding-relevant information from the SPM.mat file which is generated as part of a standard general linear model (GLM) in SPM. This information is then automatically added to *cfg*. This interface can be used if users classify not individual brain images, but use parameter estimates of single trials or runs generated by SPM (e.g., *beta maps*, see Table 1). Assuming that a user created the SPM.mat file with unique names for all regressors (e.g., “left” and “right” for regressors related to button presses) and would like to carry out a “standard” leave-one-run-out cross validation scheme with two categories, a complete decoding analysis in TDT can be executed in only one line of code (we will explain the meaning of this below). In short, the example call


decoding_example(’searchlight’,’left’,
  ’right’,beta_dir,output_dir, 4)


will perform a cross-validated leave-one-run-out searchlight decoding analysis between the regressors “left” and “right,” where *beta_dir* is the path where the SPM GLM results are stored, *output_dir* is the folder where the decoding results will be saved, and 4 is the radius of a spherical searchlight in voxels (see next paragraph for a detailed explanation). This analysis will yield a map of accuracies that can be inspected with SPM or other third-party software and which should uncover brain regions involved in encoding the motor response.

**Figure 2:**
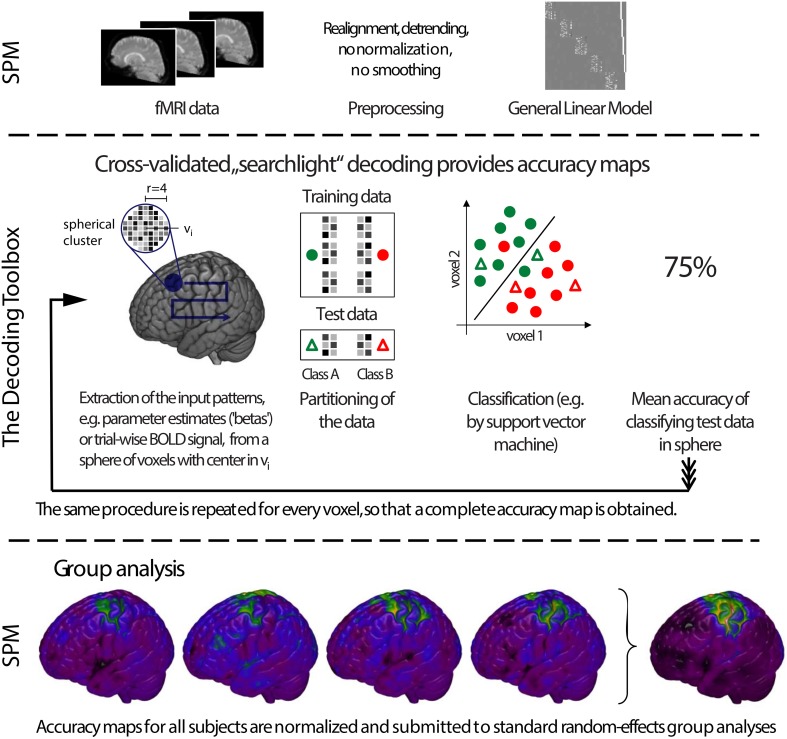
**General analysis stream of a typical searchlight decoding analysis in The Decoding Toolbox (TDT). Top**: Typical preprocessing of data is done prior to running TDT, for example with the common software SPM. Rather than submitting individual images to decoding analyses, it has become quite common to use temporal compression or statistical estimates of trials (trial-wise decoding) or of multiple trials within one run (run-wise decoding) as data for classification. **Middle**: The decoding stream of cross-validated searchlight decoding. After selecting voxels from a searchlight and extracting data from the preprocessed images, a leave-one-run-out cross-validation is performed. In this, data is partitioned, where in successive folds data from one run is used for testing and data from all other runs for training. In each fold, a classifier is trained and its performance is validated on the left-out test set. Finally, the performance of the whole cross-validation is evaluated, typically by calculating the mean accuracy across all cross-validation iterations. The accuracy is then stored at the center of the current searchlight. The whole procedure is repeated for all voxels in the brain, yielding a complete map of cross-validation accuracies. **Bottom**: Usually, these searchlight maps are post-processed using standard random effects analyses, for example using SPM's second-level routine.

To allow a first look inside the “black box,” we will now explain the inner functions of this example call (Figure 1). Prior to running a decoding analysis, TDT assumes that data have been preprocessed appropriately (Figure 2 top). Standard preprocessing includes spatial realignment, possibly slice timing correction and detrending, and in some cases also spatial normalization and smoothing (although these latter steps are less common for preprocessing of decoding analyses). As mentioned above, rather than running a decoding analysis on individual images, it has become quite common to use single trial estimates of data (Mumford et al., 2012) or even estimates that combine multiple trials within one run (Haynes et al., 2007). This process can improve classifier performance (Mourão-Miranda et al., 2006, 2007; Mumford et al., 2012), and combining multiple trials can lead to higher classification accuracies (Ku et al., 2008) and slightly improved power (Allefeld and Haynes, 2014). These estimates would typically be generated in the common GLM framework and are stored as so called beta images. For simplicity, in the following we will assume that one beta image per condition per run is generated (e.g., one for “left” for run 1, one for “right” for run 1, one for “left” for run 2, one for “right” for run 2, etc.). In this case, the above example call simply gets the beta images from *beta_dir* representing the conditions “left” and “right” and will extract the run numbers. The brain mask which SPM automatically creates during model estimation contained in *beta_dir* is automatically used to reduce the analysis to voxels inside the brain. This information is sufficient to carry out a decoding analysis with a leave-one-run-out cross-validation scheme (see Table 1 for terminology), and in this example, a so-called searchlight analysis is executed with a radius of 4 voxels, using a support vector machine (SVM, Cortes and Vapnik, 1995) as a classifier. On a Dell Precision M4600 (Intel Core i7-2820QM @ 2.30 GHz, 64 bit Windows 7) with Matlab (2011a), this analysis takes roughly 3 min for around 120,000 searchlights.

For a group analysis, this procedure is repeated for every participant, and the resulting accuracy maps can then be spatially normalized and submitted to standard statistical analysis procedures in SPM or any other preferred software package (Figure 2 bottom).

## A closer look at the decoding toolbox—for intermediate level users and above

Although the most basic one-line-of-code use of the toolbox already allows running a multitude of analyses with practically no programming skills, users may want to adjust a number of settings. In this section, we explain the major steps that are required in each decoding analysis (Figure 1B), and in the next section optional steps are explained. For each of these required steps there are default settings in TDT, but the user may wish to adjust them. In general, each user creates a short script which contains (1) all the settings of the *cfg* variable and (2) possibly the automatic extraction of data from an existing SPM model.

Basic structure:


cfg = decoding_defaults(); % optional:
    initializes cfg with default values
    ... % additional lines that modify cfg
    might go here
results = decoding(cfg); % Performs
    decoding


Concrete example call:


cfg = decoding_defaults();
regressor_names = design_from_spm
   (beta_dir); % beta_dir is directory
   of SPM model
cfg = decoding_describe_data
   (cfg,{’left’,’right’},[1 −1],...
   regressor_names,beta_dir);
   % 1-1 are arbitrary label numbers
   for red & green
cfg.design = make_design_cv(cfg);
results = decoding(cfg);


This example automatically extracts regressor names from the SPM model, then accumulates all relevant information from the design related to the regressors of interest (“left” and “right”), creates a leave-one-run-out cross-validation design, and executes the decoding analysis using default settings. Important basic settings that otherwise use these default settings are explained in the next four paragraphs.

### Design creation

**Figure 3:**
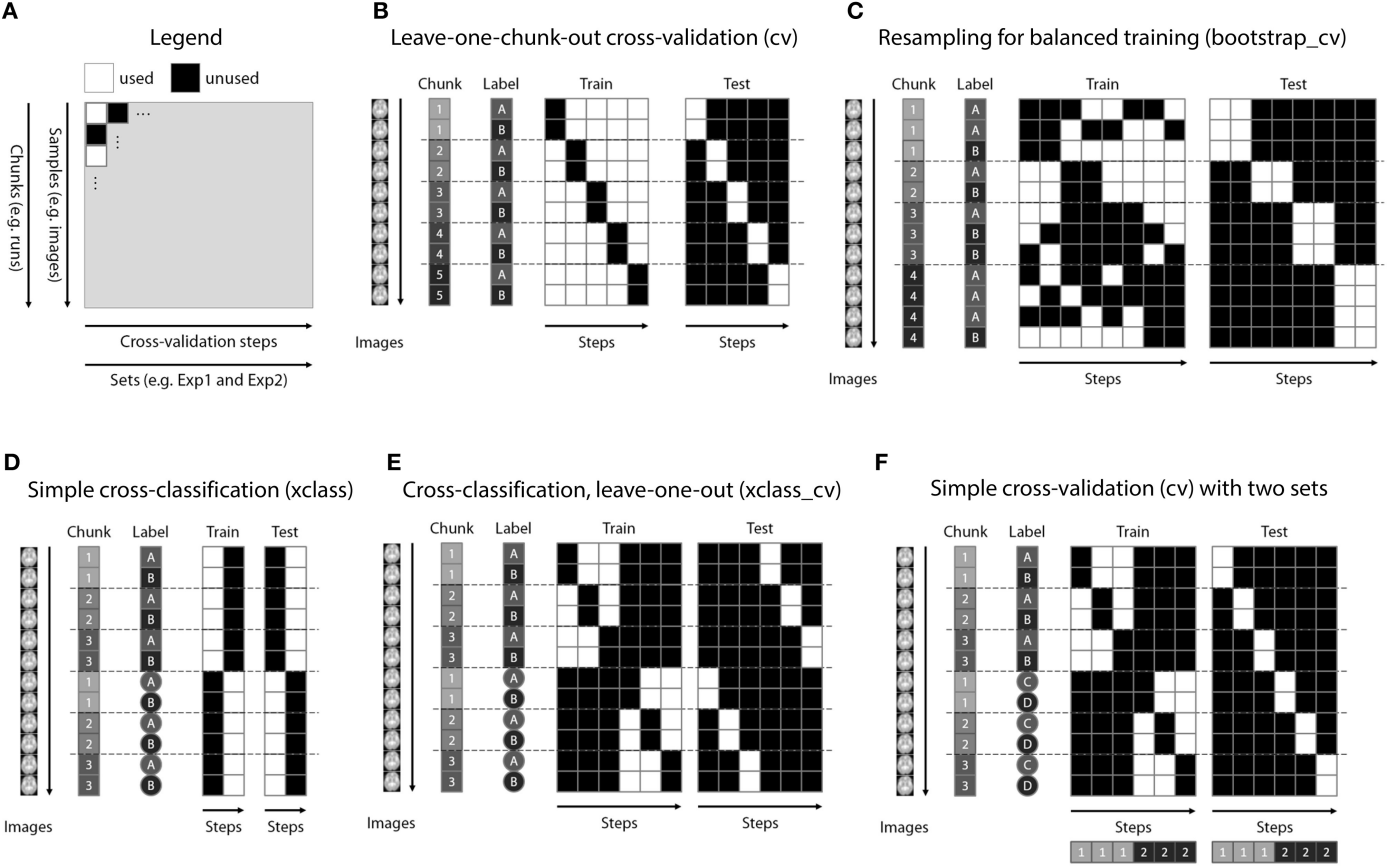
**Decoding design matrices. (A)** General structure of a decoding design matrix. The vertical dimension represents different samples that are used for decoding, typically brain images or data from regions of interest. If multiple images are required to stay together within one cross-validation fold (e.g., runs), this is indicated as a chunk. The horizontal axis depicts different cross-validation steps or iterations. If groups of iterations should be treated separately, these can be denoted as different sets. The color of a square indicates whether in a particular cross-validation step a particular brain image is used. **(B)** Example design for the typical leave-one-run-out cross-validation (function *make_design_cv*). **(C)** Example design for a leave-one-run-out cross-validation design where there is an imbalance of data in each run. To preserve balance, bootstrap samples from each run are drawn (without replacement) to maintain balanced training data (function *make_design_boot_cv*). **(D)** Example design for a cross-classification design which does not maintain the leave-one-run-out structure (function *make_design_xclass*). **(E)** Example design for a cross-classification design, maintaining the leave-one-run-out structure (function *make_design_xclass_cv*). **(F)** Example design for two leave-one-run-out designs with two different sets, in the same decoding analysis. The results are reported combined or separately for each set which can speed-up decoding.

A powerful feature of TDT is the use of decoding design matrices (Figure 3). A decoding design matrix determines the exact structure of a cross-validation design, i.e., which data belongs to which class and which data is training and test data in each *decoding step* (also called *cross-validation fold* or *iteration*). To determine which samples should remain together (e.g., because they are in one run and the analysis is a leave-one-run-out cross-validation), data can be assigned to *chunks* which serve as categorical numerical labels. Although in many cases, the number of chunks determines the number of decoding steps, more complex designs (e.g., bootstrapping designs, Figure 3C) can have more steps than chunks. Finally, multiple decoding analyses can be carried out in one design, a feature which might be particularly useful if data needs to be analyzed separately and combined, or if cross-classification is applied to a number of different data sets saving repeated training on the same data. A number of existing functions allow creating a multitude of different designs in advance, and additional design functions can be created by more advanced users (see Figure 3 for some example designs using different functions). After creating the design, the design inspection can be used to visualize if the analysis is carried out in the correct manner.

Example call (design created and visualized prior to a decoding analysis as part of the script):


cfg.design = make_design_cv(cfg);
   % creates the design prior to decoding
display_design(cfg)% visualizes the
   design prior to decoding


Alternative example call (design created and visualized while the decoding analysis is running):


cfg.design.function.name = ’make_design_cv’;
   % creates a leave-one-chunk-out
   cross-validation design
cfg.plot_design = 1;
   % plots the design when decoding starts


### Analysis type

Most decoding analyses can be categorized as one of three different types of analysis, depending on the general voxel selection criterion: Whole-brain analyses, region-of-interest (ROI) analyses, and searchlight analyses. All of these approaches are commonly used for decoding. In the machine learning community, ROI and searchlight analyses might be considered feature selection approaches, but this is not typically how they are called in the brain imaging community. All of these approaches have their respective advantages and disadvantages (discussed e.g., in Etzel et al., 2009, 2013). Among others, one advantage of whole-brain analyses is that all available information is fed into the classifier, while major disadvantages of this method are the difficulty to tell the origin of the information and the so-called “curse of dimensionality” (see Table 1). These problems are less of an issue in ROI analyses, but ROIs need to be specified and can in that way be biased, variable ROI sizes make them difficult to compare, and information encoded across different ROIs gets lost. Searchlight analyses can be seen as a succession of spherical ROIs, but require no prior selection of brain regions and are in that respect unbiased to prior assumptions about where to expect an effect (Friston et al., 2006); however, they suffer from the multiple comparisons problem, the necessity to specify the searchlight size as an additional parameter, and are—just like ROIs—insensitive to information encoded across distant brain regions.

Example call:


cfg.analysis = ’wholebrain’;
  % alternatives: ’ROI’, ’searchlight’


### Train and test classifier

The core of the toolbox is training and testing the classifier. A classifier is typically first built from data where class membership is known (“train” classifier); then, the ability of the classifier to generalize to unseen data is evaluated (“test” or “validate” classifier). This separation is important to limit the impact of overfitting the classifier to the data and to have an unbiased estimate of the classifier's generalization performance. The training and test cycle is hardcoded to make sure that training and test data are truly kept separate in each cycle (unless users create their own extension to overcome this separation, if required). TDT is equipped with a number of external classifiers which can be selected by setting the *cfg* variable. These classifiers belong to the packages LIBSVM (Chang and Lin, 2011) and LIBLINEAR (Fan et al., 2008) and include *L*1- and *L*2-norm regularized support vector machines and logistic regression. In addition, a correlation classifier (Haxby et al., 2001) has been implemented as part of the toolbox. Multiple classifiers have been compared across different data sets, with variable results (Cox and Savoy, 2003; Pereira et al., 2009; Misaki et al., 2010). Typically, the *L*2-norm support vector machine (SVM) performs quite well, which is why it is the default classifier in TDT. The options of the classifiers can be set using the *cfg* variable. TDT can also be used for purposes other than classification, for example to conduct a simple correlation of two data sets. Finally, at the stage of training and testing, advanced users can create an interface to completely external toolboxes that include other optimization methods (see How to Extend the Toolbox). All input can be passed as part of the settings in the *cfg* and all output can be stored for later processing.

Example call:


cfg.decoding.software = ’liblinear’;
cfg.decoding.method = ’classification’;
cfg.decoding.train.classification.
  model_parameters = ’-s 0 -c 1 -q’;
cfg.decoding.test.classification.
  model_parameters = ’-q’;


### Transformation of results

Classifiers are essentially functions that describe a separation boundary (i.e., hyperplane, see Table 1) of multiple classes in voxel space. Newly predicted samples are mapped relative to this function and typically receive an output larger or smaller than 0 that denotes the distance to the separating function, where positive values denote membership to one class and negative values membership to the other class. For linear classifiers, so called decision values can be used to indicate the distance of a particular sample to the separating hyperplane. Using the predicted labels, the actual labels and the decision values, a number of metrics can be calculated to evaluate the performance of a classifier.

The most typical output for a decoding analysis is the mean cross-validated accuracy value. However, a number of other types of output are quite common and potentially useful. For example, it can be of interest to look at the classification accuracy for each class separately to assess classifier bias. A classification accuracy of 75% should be interpreted very differently when either class is decoded with 75% accuracy, or when one is decoded with 100% accuracy and the other with 50% (leading to 75% on average for a balanced number of samples in each class). In case of differently sized classes, balanced accuracy or d-prime can give indices of performance that take into account the different size of the groups. Additionally, the area under the receiver-operator-characteristic curve (AUC) which uses the distance of a classification output to the decision boundary can provide results about the information content using a graded rather than a binary response.

Other measures have received interest as well. For instance, weight maps are useful to provide information about the contribution of each voxel to the classification and are often used in whole-brain classification settings. Importantly, weight maps cannot be interpreted as reflecting the neural substrate relating to a task or another variable that is classified. Researchers interested in interpreting weights beyond the classifier alone can convert the weight maps to patterns which then show the contribution of each voxel for the representation of the classes under study (Haufe et al., 2014; Ritter et al., 2014). In addition, representational similarity analysis which exploits the correlation similarity structure of voxels and different distance metrics can be used to illustrate the representational distance of different conditions (Kriegeskorte et al., 2008; Nili et al., 2014). All approaches described above have been implemented in TDT.

Example call:


cfg.results.output = {’accuracy_minus_
  chance’, ’AUC_minus_chance’,
  ’SVM_weights’};
cfg.decoding.method = ’classification’;
  % weights cannot be extracted when
  precomputed kernels are used, i.e.,
  we need to use the slower method of
  classification without kernels


### Statistical analysis

For a neuroscientist, it is important to statistically evaluate the results generated by a classification analysis: Is the observed result statistically significantly different from chance? Although at a first glance a decoding accuracy close to chance may seem disappointing, it is indeed quite commonly observed and not necessarily a problem. In neuroscience, typically the goal is not to maximize classification performance, but to demonstrate that information is present in the brain signals used for classification. This contrasts with the use in machine learning where the primary goal is to maximize classification performance and accuracies only slightly above chance would not be considered very useful. For that reason, there is not much work investigating the combination of multivariate decoding with classical statistics.

Importantly, it can be shown that combining results based on cross-validation and classical statistics can lead to overly liberal results. For example, cross-validated decoding accuracies are not binomially distributed, which is why binomial tests should not be used to statistically evaluate cross-validation results (Schreiber and Krekelberg, 2013; Noirhomme et al., 2014). As an alternative, permutation testing can be used. However, for searchlight analyses this can become very time consuming. In addition, caution is warranted when using permutation approaches to make sure that these tests are not overly liberal (Stelzer et al., 2013), for example because they break up statistical dependencies at the wrong level (i.e., they violate the assumption of exchangeability, Nichols and Holmes, 2002).

These problems relate only to statistical analyses at the “decoding-level.” An alternative and common approach is to combine multiple decoding results and conduct a “second-level” statistical analysis. This is the case when evaluating whether the mean accuracy across a group of subjects is different from chance. For these purposes, classical parametric tests such as the Student's *T*-test can be used.

In TDT, at the decoding-level both binomial testing and permutation testing have been implemented. However, binomial testing should only be used when training and test data are not reused, i.e., when no cross-validation and no bootstrapping is performed. A permutation test is conducted in three steps: First, the same decoding design is set up using a permutation scheme where data from different chunks is kept separate. Second, the permutations are conducted which can be run in parallel to speed-up processing. Third, the permutation test is conducted. Results can be reported in Matlab or written to brain images. For the second-level, users are advised to use dedicated third-party software such as SPSS, R, or Matlab which is specialized for statistical analyses and allows testing for basic assumptions of the tests. For users who do not want to export their results to other packages and are confident that the assumptions of their tests are met, TDT offers a set of basic functions that can conduct classical *T*-tests and *F*-tests.

Example call:


[results,cfg] = decoding(cfg);
   % run previously prepared decoding
cfg.design = make_design_permutation
   (cfg, 1000, 1); % creates one design
   with 1000 permutations
[reference,cfg] = decoding(cfg);
   % run permutations
cfg.stats.test = ’permutation’; % set test
cfg.stats.tail = ’right’;
   % set tail of statistical correction
cfg.stats.output = ’accuracy_minus_chance’;
   % choose from all original outputs
p = decoding_statistics
   (cfg,results,reference);


## A description of optional workflow steps—for advanced level users

This section describes all remaining classification-related methods that can be carried out using a decoding analysis in TDT (Figure 1C). These include feature scaling, selection of model parameters in nested cross-validation, transformations of feature space, and feature selection. None of these steps are strictly necessary for a decoding analysis, but they can in principle help improving the results of a decoding analysis.

### Scaling

Scaling is the process of adjusting the range of data which enters the classifier. This can be done to bring data to a range which improves the computational efficiency of the classifier (for example LIBSVM recommends scaling all data to be between 0 and 1). It can, however, also be used to change the relative contribution of individual features or individual samples or to remove the influence of the mean spatial pattern (Misaki et al., 2010; but see Garrido et al., 2013) which might affect classification performance. Scaling is also known as normalization, but we prefer the term scaling to distinguish it from another meaning of the term “normalization” which is commonly used in the MRI community to refer to spatial warping of images.

Typically, row scaling is used, i.e., scaling across samples within a given feature. Although scaling can theoretically improve decoding performance, for some data sets it may not have any influence (Misaki et al., 2010). Practically, scaling often has little or no influence on decoding performance when beta images or z-transformed data are passed, because this data already represents a scaled form of the raw images which is scaled relative to each run, rather than to all training data. However, scaling may still speed-up classification.

TDT allows a number of different settings: Either all data are scaled in advance (in TDT: “all”), which is only valid when scaling carries no information about class membership that influences test data, or scaling is carried out on training data only and these estimated scaling parameters are then applied to the test data (in TDT: “across”). The typically used scaling methods which have also been implemented in TDT are min0-max1 scaling or z-transformation. Min-max scaling scales all data to a range of 0 and 1, while z-transformation transforms data by removing the mean and dividing by the standard deviation. In addition to scaling data to a specified range, cut-off values can be provided for outlier reduction (Seymour et al., 2009). With this setting, all values larger than the upper cut-off are reduced to this limit, and all values smaller than the lower cut-off are set to this value. In TDT, these approaches can be combined with outlier reduction.

Example call:


cfg.scale.method = ’across’;
   % scaling estimated on training data and
   applied to test data
cfg.scale.estimation = ’z’;
   % z-transformation as scaling approach
cfg.scale.cutoff = [-3 3]; % all values
   > 3 are set = 3 (here: 3 standard
   deviations, because data is
   z-transformed)


### Parameter selection

Most multivariate classification and regression approaches use algorithms that contain parameters that need to be set by the user. For example, linear support vector machines have the regularization parameter *C* that determines the degree to which data points can cross the so-called *margin* (Table 1) which can even lead to misclassification during training (Cortes and Vapnik, 1995). Large values of *C* strongly penalize misclassification in training while small values of *C* allow for larger margins. This variable influences the bias-variance tradeoff, i.e., the tradeoff between fitting a classifier too close to the training data (overfitting), or fitting it too little (underfitting, i.e., “ignoring” the structure of the training data too much), which directly affects the ability to generalize to the test data (Müller et al., 2001). Another example is the parameter *gamma* that needs to be set for non-linear radial basis function (RBF) SVMs which determines the width of the RBF kernel (Schölkopf et al., 1995). Since the optimal values for *C* and *gamma* are not known in advance, they are often estimated on the training data using *nested cross-validation*, a method where a cross-validation scheme is used only within training data to find good parameters. Typically, all parameter combinations across a range of target values are tried, a process known as *grid search*. The parameter combination that leads to best classification performance within training data is then applied to all training data and validated on the left-out test data. This has the consequence that for each real cross-validation fold a different set of optimal parameters might be chosen.

To avoid nested cross-validation, many researchers simply choose to set *C* to a fixed value of 1. In the typical scenario of an SVM where the number of features (e.g., voxels) is much larger than the number of samples (e.g., trials or beta images), in our experience only *C*-values much smaller than 1 have any influence on classification. This is probably because all samples within training data can easily be linearly separated, and for larger values of *C* the classifier yields stable solutions. Another problem is that with a small number of samples—even with nested cross-validation—the estimated parameters become quite variable, because they fit to the specifics of the training data and might not generalize well to test data. In particular cases, this might improve decoding performance slightly. Importantly, researchers should bear in mind that they must not escape the idea of cross-validation by trying out multiple parameter combinations and picking the one that yields the “best” results in the final cross-validated classification accuracy maps. Rather, they should use nested cross-validation for picking the optimal parameter and testing whether this choice generalizes to independent test data in the outer cross-validation loop. Alternatively, they could use parameters based on (previous) independent data sets or independent contrasts. Finally, the disadvantage of parameter selection is that it is quite time consuming, in particular when multiple parameters are selected concurrently. Parameter selection might make more sense in other circumstances or when other parameters are selected, for example the degree of a polynomial kernel.

In TDT, parameter selection is currently implemented as grid search, where all parameter combinations are combined. Depending on the problem, there might be smarter optimization approaches, but in our experience grid search works well for optimizing few parameters, in particular when only few samples are available. More advanced users can also create their own functions to tailor parameter selection to their specific problem.

Example call:


cfg.parameter_selection.method = ’grid’;
  % grid search (currently the only
  implemented method)
cfg.parameter_selection.parameters
  = {’-c’,’-g’};
cfg.parameter_selection.parameter_range =
  {[0.0001 0.001 0.01 0.1 1 10 100 1000];
  [0.5 1 2]};
% The following parameters are set as
  defaults, i.e., they don’t need to be
  called explicitly
cfg.parameter_selection.format.name =
  ’string_number’;
cfg.parameter_selection.format.
  separator = ’ ’;
cfg.parameter_selection.optimization_
  criterion = ’max’; % pick value with
  maximal accuracy


### Feature transformation

The term *feature transformation* is not commonly used in the brain decoding community, but it is known in the machine learning community as a set of methods that change the meaning of each individual feature (Liu and Motoda, 1998). We use this term to refer to a collection of methods that is used to change the space of data and possibly reduce the dimensionality of this changed space, as well. Principal component analysis (PCA) is one example. We prefer to treat such methods separately from *feature selection* (see below), because they lead to a change in the mapping of features. Methods that we refer to as *feature selection* (below) select a subset of features (e.g., voxels) of the input. In contrast, in *feature transformation* a data point refers to a non-linear transformation applied to the input, which essentially creates new features. For example, the outcome of a principle component analysis (PCA) is no longer separate single voxel data, but a linear combination of the original voxel data. This strict semantic separation helps to avoid confusion, makes the results of feature selection easier to interpret, facilitates the addition of new methods, and facilitates the use of multi-step feature selection (e.g., first running PCA and transforming voxels to principle component space, and then running feature selection on a subset of principal components). Other examples of feature transformation would encompass independent component analysis (ICA), Fourier transformation, or pre-calculated mappings that can for example be used to align data spaces between subjects, known as hyperalignment (Haxby et al., 2011). Currently, only PCA is implemented in TDT, but other methods can easily be added. The transformation can be estimated on training data only and applied to test data in each cross-validation step (“across”). Alternatively, it can be estimated on both training and test data (“all”) when it is ensured that this does not lead to non-independence (Kriegeskorte et al., 2009). This should not be the case as long as no label information is used for transformations (which is not done in PCA). If feature transformation is also used for dimensionality reduction, the user can specify either the number of to be selected features or can specify a critical value in the score of the method that needs to be exceeded for a transformed feature to be of interest (e.g., for PCA the percent variance explained).

Example call:


cfg.feature_transformation.method = ’PCA’;
cfg.feature_transformation.
    estimation = ’all’;
cfg.feature_transformation.critical_value =
    0.1; % only keep components that
    explain at least 10 percent variance


### Feature selection

Books have been written about *feature selection* (Guyon et al., 2006) which is a method in machine learning that refers to the reduction of the dimensionality of data, with the aim of finding the most relevant features and for improving classification performance. Feature selection has been classified into three general categories: filters, wrappers, and embedded methods (Guyon and Elisseeff, 2003). Filters are methods that use univariate or multivariate statistics based on the data to rank features and select them based on their rank. Examples are the F-statistic, the weights of a classifier, or in brain imaging external masks that provide a ranking, such as external functional localizer images. Wrappers are methods that iteratively include or exclude features, based on some optimization criterion specific to the feature selection method. Examples are sequential forward selection or sequential backward elimination. Finally, embedded methods are methods where the selection of features becomes part of the classification problem. Examples are LASSO (Tibshirani, 1996) or *L*1-regularized support vector machines (Fan et al., 2008) where unimportant voxels receive a weight of zero, which eliminates their contribution. Recursive feature elimination is another popular method (De Martino et al., 2008) and is sometimes referred to as a wrapper method, although the final feature set and classification can depend on the previous steps. For that reason—and to better distinguish it from other sequential methods—it can also be referred to as an embedded method.

In TDT, feature selection has been implemented with a number of filter methods and with the embedded method of recursive feature elimination. Implemented filter methods are the F-statistic, the Mann-Whitney U-statistic, classifier weights, and external masks, with the additional option of supplying a separate mask for each cross-validation fold. For determining the optimal feature set, nested cross-validation can be performed. It should be noted, however, that similar to parameter selection the utility of feature selection is limited when the number of samples is very small. In that case, nested cross-validation can optimize the number of features to the idiosyncrasies of the data, and it might be better not to perform feature selection at all. In addition, feature selection can be computationally highly expensive and can thus dramatically slow down classification, in particular when recursive feature elimination is used with nested cross-validation and a large number of steps. As has been mentioned for parameter selection, researchers should be cautious not to try out a number of different feature selection methods and choose one that produces the “best” final result. Often researchers might be interested in implementing multiple feature selection steps. In the current version of TDT, up to two feature selection steps are possible. More sophisticated feature selection methods can be added to TDT, e.g., by including them directly into custom classification routines.

Example call:


cfg.feature_selection.method = ’filter’;
cfg.feature_selection.filter = ’F’;
cfg.feature_selection.n_vox = ’automatic’;
   % nested CV to determine optimal number
   of features


More sophisticated example call:


% First, run feature selection using
   external ranking mask
cfg.feature_selection.feature_selection.
   method = ’filter’;
cfg.feature_selection.feature_selection.
   filter = ’external’;
cfg.feature_selection.feature_selection.
   external_fname = ’mylocalizer.img’;
cfg.feature_selection.feature_selection.
   n_vox = 0.5; % select 50% of all voxels
% Then run Recursive Feature Elimination on
   the remaining voxels
cfg.feature_selection.method = ’embedded’;
cfg.feature_selection.embedded = ’RFE’;
   % Recursive Feature Elimination
cfg.feature_selection.n_vox =
   [5 10 20 40 80 160]; % possible target
   values for number of voxels in RFE
   (i.e., where RFE may stop in the end)
cfg.feature_selection.nested_n_vox =
   [5:5:200]; % eliminate 5 voxels per
   step in nested CV


## How to extend the toolbox

TDT comes with a number of processing approaches, but of course not all methods have been implemented. Researchers might want to use their preferred machine learning algorithm or feature selection method for their decoding analysis. Other users might wish to use TDT only as a wrapper tool for their own machine learning package. Data can also be passed to TDT, which in principle extends TDT to other modalities such as EEG or MEG decoding. In this section, we will use three examples to illustrate how TDT can be extended.

### Example 1: introduce a new classifier

To add new classifiers, two functions need to be provided, one for training and one for testing the classifier. If the new classifier should be called “newclassifier,” the training method needs to be saved as newclassifier_train.m and is called by


model = newclassifier_train
    (labels_train,data_train,cfg)


where *model* is any information that is needed to evaluate the classifier. Within the function, the *cfg* is needed to distinguish whether a classification or regression should be performed, or whether a precomputed kernel is passed. In addition, the parameters for classification can be passed in this variable. In the following, we show pseudocode for the function to implement a new classifier.


function model = newclassifier_train
     (labels_train,data_train,cfg)
switch lower(cfg.decoding.method)
case ’classification’
model = your_train_algorithm(labels_train,
    data_train,cfg.decoding.train.
    classification.model_parameters)
case ’classification_kernel’
    ...
case ’regression’
    ...
otherwise
   error(’...’)
end


If the external function creates several outputs, these can be grouped within the variable *model*. The structure for the second function parallels the first and looks as follows:


function decoding_out = newclassifier_test
   (labels_test,data_test,cfg,model)
switch lower(cfg.decoding.method)
case ’classification’
[predicted_labels decision_values
   other_output] = your_test_algorithm
   (labels_test,data_test,model,
   cfg.decoding.test.classification.
   model_parameters);
decoding_out.predicted_labels =
   predicted_labels;
decoding_out.true_labels = labels_test;
decoding_out.decision_values =
   decision_values;
decoding_out.model = model;
decoding_out.opt = other_output;
   % optional other output which we added
   just as an example
(...)
end


It is important that *decoding_out* returns a struct variable (i.e., structure array) with the subfields above (predicted_labels, true_labels, decisions_values, model, opt). If some of these are not provided by a classifier, empty arrays can be passed. These new classifier functions can then be called in any decoding analysis by setting


cfg.decoding.software = ’newclassifier’;


### Example 2: introduce a new result measure

New, more sophisticated means of transforming results can also be introduced to TDT. For example, consider a user who wants to weight classification accuracies by decision values before averaging. The function would be written as follows:


function output = transres_dvtimesacc
    (decoding_out,chancelevel,cfg,data)
predicted_labels = vertcat
    (decoding_out.predicted_labels);
true_labels = vertcat
    (decoding_out.true_labels);
accuracy = predicted_labels==true_labels;
decision_values = vertcat
    (decoding_out.decision_values);
dv_norm = abs(decision_values)/
    max(abs(decision_values));
output = 100 * accuracy.* dv_norm;


The function should be saved as transres_dvtimesacc.m and can then be used by setting


cfg.results.output = ’dvtimesacc’;


### Example 3: extend toolbox to external machine learning package or more complex processing streams

The toolbox can be interfaced with complete external machine learning packages, for example, the powerful Shogun toolbox (Sonnenburg et al., 2010). This might be useful for users who want to use the general workflow of TDT, e.g., the data handling, cross-validation facilities and searchlight routines, but do not wish to implement a large number of external algorithms for use in TDT. The same general procedure can be applied to use highly sophisticated processing streams that do not directly fit into the TDT framework. There are two approaches for creating an interface to an external toolbox. The first takes place at the level of the classifier. Essentially, an interface is created where all steps of the decoding analysis—if requested, even including cross-validation—are carried out outside of TDT. The training function of a classifier acts as a placeholder to pass data to the testing function.


function model = external_toolbox_train
    (labels_train,data_train,cfg)
model.data_train = data_train;
model.labels_train = labels_train;


The interface function would look as follows:


function decoding_out =
    external_toolbox_test
    (labels_test,data_test,cfg,model)
[predicted_labels accuracy decision_values]
    = externalpackagewrapper(data_train,
    labels_train, data_test,
    labels_test,cfg)


The function *externalpackagewrapper* would then contain the script which is normally executed using the external software package. This can be made general purpose by passing parameters using the setting *cfg.decoding.train.classification.model_parameters* of TDT.

The second approach for creating an interface to external toolboxes takes place at the level of the transformation of outputs. This might be useful if a user would like to complete the entire decoding analysis including the generation of output in an external toolbox. In this case, the user creates a placeholder for the classifier which merely passes all data and classification parameters inside the *model* variable as the optional output *decoding_out.opt* (e.g., using the toolbox function “passdata” as a classifier). Then, the interface to the external package is created as follows:


function output = transres_packageinterface
    (decoding_out,chancelevel,cfg,data)
output = externalpackagewrapper
    (decoding_out.opt,data)


where *output* contains all desired output of the external package.

## Toolbox validation: simulations and applications to fMRI data

To demonstrate that TDT works correctly, we validated the toolbox by running a number of analyses on simulated and real data. These analyses are not supposed to provide an overview over all capabilities of the toolbox. Rather, they are used to illustrate some of the functionalities of TDT.

### Simulations: searchlight analysis, whole-brain analysis, region-of-interest analysis

**Figure 4:**
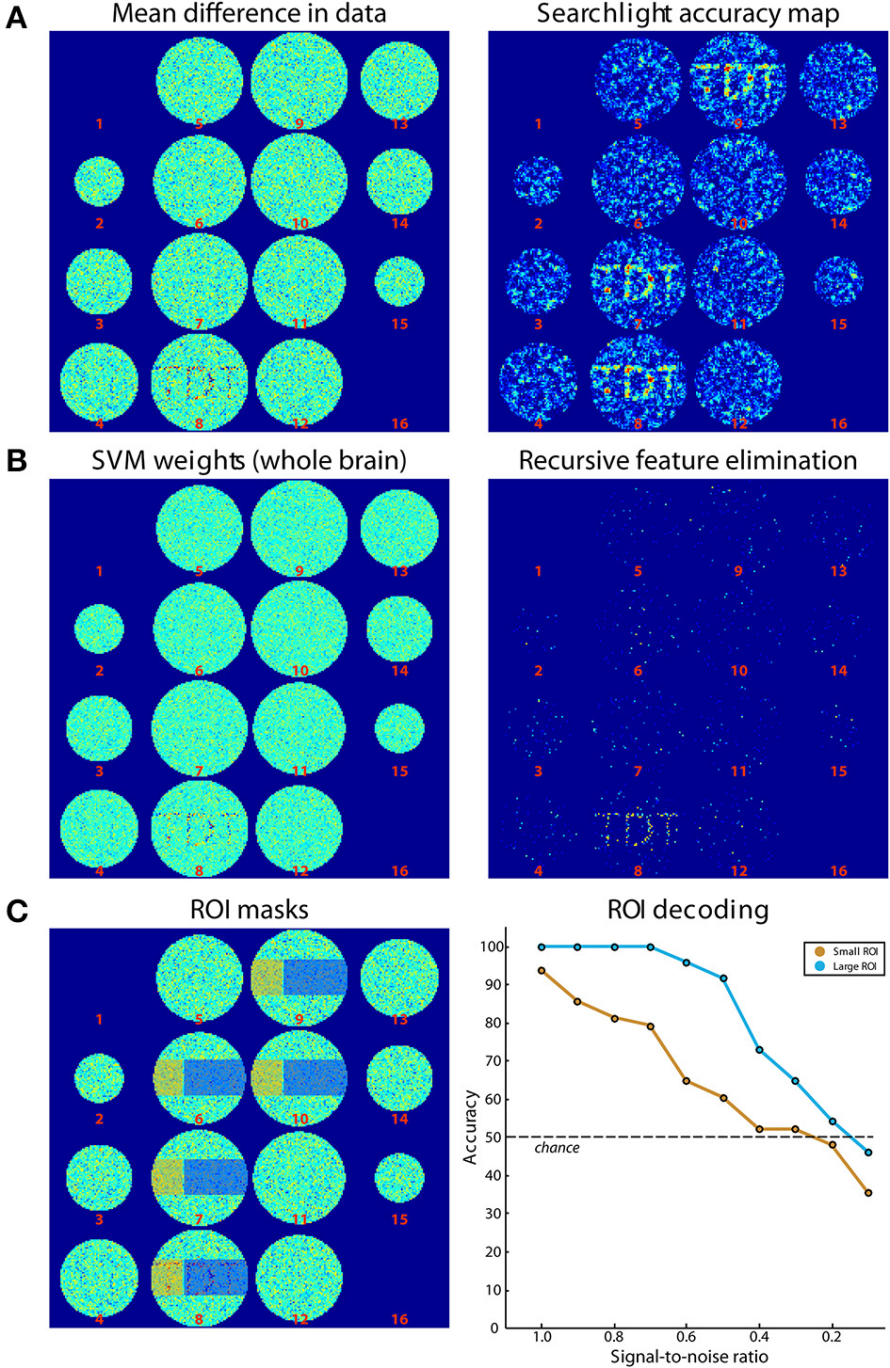
**Results of simulations. (A)** On the left, the difference of the mean of images belonging to both classes is shown. On the right, the results from Simulation 1 (searchlight analysis) are plotted. **(B)** Results from Simulations 2A and 2B. The left panel shows the weights of the SVM trained on data in **(A)**. On the right, the results from a recursive feature elimination are plotted. **(C)** Results from Simulation 3. The left panel shows the ROIs that were selected. The right panel shows decoding accuracies in the two ROIs depending on the SNR.

Simulated data consisted of 48 ellipsoid volumes (31,016 voxels), centered in volumes of 64 × 64 × 16 voxels (Figure 4). Each voxel was assigned independent Gaussian random noise (mean = 0, standard deviation = 1). Half of the volumes were assigned to the “noise” category and remained unchanged. The other half was assigned to the “signal” category where a pattern was added to axial slice 8. This pattern consisted of Gaussian random noise (mean = 0, standard deviation = 0.8). The same pattern was added to all signal volumes. Data was then split in 8 runs of 6 volumes each, providing three samples per category in each run. Please note that noise is spatially and temporally independent, which is a strong simplification but is sufficient for the purpose of this simulation. Additionally, univariate effects are present which is not necessary for classification to work. The difference of the mean results is shown in Figure 4A (left panel).

For Simulation 1, we ran a searchlight analysis with a leave-one-run-out cross-validation scheme. The searchlight had a radius of 2 voxels, encompassing 27 voxels. The classifier was a linear L2-norm SVM with *C* = 1, in the implementation of LIBSVM (Chang and Lin, 2011). As output, the mean classification accuracy was returned. The results are shown in Figure 4A (right panel). As expected, the accuracies around the signal regions are highly above chance while in other regions they fluctuate around chance-level. In addition, Figure 4A (right panel) illustrates the slight smoothing imposed by the searchlight, where the high SNR spreads the word “TDT” to the neighboring axial slices (Etzel et al., 2013).

Simulation 2 consisted of two whole-brain analyses that were run on the same simulated data set used for Simulation 1. In Simulation 2A, we used all data to generate a weight map, i.e., we did not separate data in training and test sets. A weight map indicates the contribution of each voxel to the classification which reflects a combination of signal enhancement and noise suppression. As can be seen in Figure 4B (left panel), the results are similar to the difference in means of the original data, because noise was spatially uncorrelated. Simulation 2B used the same leave-one-run-out cross-validation scheme as in the searchlight analysis to achieve one classification accuracy for the entire brain. The analysis consisted of an additional nested cross-validation where we conducted recursive feature elimination to identify the set of voxels that is optimally suited for carrying out the classification task. Classification performance was at ceiling (100 % accuracy). Figure 4B (right panel) shows how often each voxel is chosen in any of the six cross-validation steps for recursive feature elimination, again clearly favoring signal voxels over noise voxels.

In Simulation 3, we ran a region-of-interest (ROI) analysis with two ROIs, where one ROI covered one third of the signal region while the other covered the remaining two thirds (Figure 4C, left panel). Again, we used a leave-one-run out cross-validation scheme. We then continuously varied the amount of signal that was added to the noise. As can be seen in Figure 4C (right panel), the decoding accuracy gradually decreased with decreasing SNR until it reached chance-level when no signal is present. In addition, the accuracy in the smaller ROI was generally lower than that in the larger ROI, reflecting the reduced amount of signal present in that region.

### Validation on empirical data: the Haxby 2001 dataset

To validate the toolbox on real imaging data, we used data from a study by Haxby et al. (2001) which is publicly available (http://data.pymvpa.org/datasets/haxby2001/). Because of space limitations, we do not use the dataset provided with the toolbox for this article, because it would require more detailed explanation of the experimental paradigm. Haxby et al. (2001) investigated processing of images belonging to 8 different object categories in ventral visual cortex. Data from six subjects were made available. For a complete description of the experimental paradigm and imaging parameters, see the original publication.

The experiment consisted of 12 runs where each category was shown once in each run in a 24 s block. Data were motion corrected and detrended using SPM12 (http://www.fil.ion.ucl.ac.uk/spm/software/spm12/). We fitted each block with a canonical hemodynamic response function, yielding one beta image per condition per run and a total of 96 beta images per subject.

**Figure 5:**
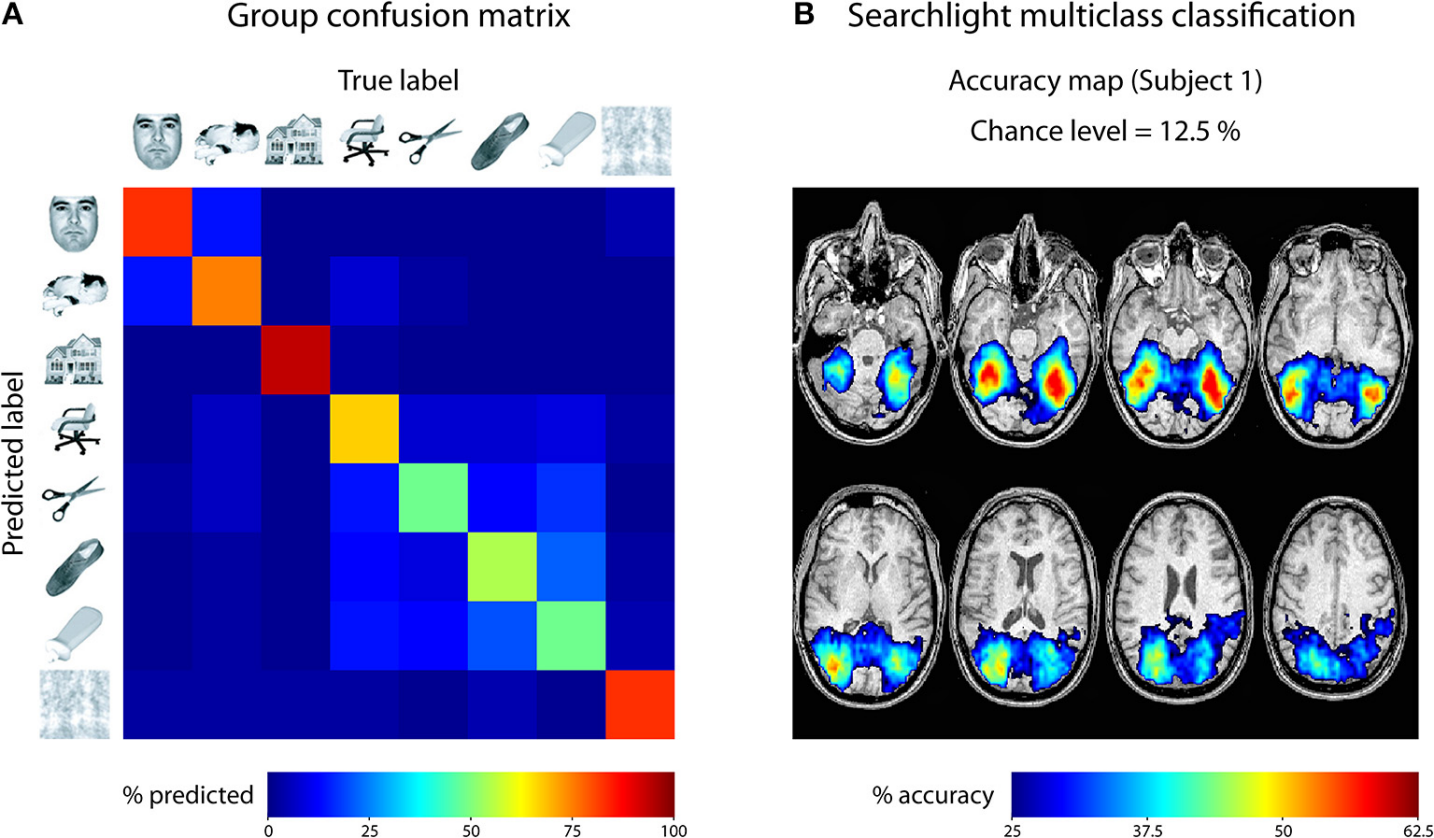
**Results of analyses on Haxby 2001 data set. (A)** Confusion matrix reflecting the confusion of all eight classes in ventral temporal cortex, averaged across all 6 subjects. **(B)** Searchlight multiclass classification results of subject 1 (permutation *p* < 0.001, cluster-corrected).

For the first empirical analysis, we ran a ROI decoding in ventral temporal cortex. Our goal was to investigate the classification accuracy and the confusion of the different categories. For that purpose, we used the masks provided with the Haxby data set to focus our analysis on ventral temporal cortex. The beta images were then submitted to a leave-one-run-out cross-validation scheme, using a one-vs.-one multiclass SVM classification approach^2^. The output was specified to reveal a confusion matrix comparing the frequency of the predicted label with the true label. This approach was repeated for all six subjects and the resulting confusion matrices were averaged. The result is shown in Figure 5A. As can be seen, each class could be classified very well, with only little confusion between the classes. The results indicate two clusters with larger confusion: Images of faces and cats were confused more often than other categories, and images of inanimate objects were confused more often. This could indicate that in ventral visual cortex, faces and cats as well as different inanimate objects are processed more similarly to each other.

For the second empirical analysis, we ran a multiclass searchlight analysis on subject 1 from the data set, with a leave-one-run out cross-validation scheme and a searchlight radius of 4 voxels. To assess statistical significance, we ran a permutation test, yielding a critical cut-off of 25 % accuracy (chance-level: 12.5 %) and a cluster size of *k* = 24 voxels (cluster-level corrected *p* < 0.001). The resulting accuracy maps were masked by the cut-off and are shown in Figure 5B. Large portions of the dorsal and ventral visual cortex carry information about the different categories, with decoding accuracies reaching 62.5 % ventral temporal cortex of subject 1. Taken together, these results fit nicely with the known architecture of the visual cortex and reflect the feasibility of the toolbox to reveal information about the content of representations in the human brain.

## Discussion

We hope that the above illustration of TDT demonstrated the simplicity and general utility of this toolbox for multivariate analysis of functional MRI data. In addition, the extensive error checking helps prevent many programming errors and should guide users in how to quickly resolve them. We did not go into detail about an additional important feature of TDT that proved helpful to prevent errors: the visualization of the decoding design. It shows users at a glance if the cross-validation design they intended to set-up is indeed the one that is computed and if the input data is indeed the data they wanted to use. This further facilitates the prevention of unwanted or erroneous analyses. To help in getting started, TDT comes with an example dataset an example analysis scripts.

### Features not contained in TDT: preprocessing and results visualization

When we developed TDT, we initially used it as a tool for conducting searchlight analyses on preprocessed data in order to create individual searchlight accuracy maps that could then be used as input to group-level analyses. Later, the toolbox was extended to become more general purpose, adding whole-brain and ROI analyses and more and more machine-learning related utilities. Our focus for TDT was on specifically creating a tool that provides users with the means to conduct multivariate decoding. Most decoding analyses are carried out on preprocessed data (e.g., spatially realigned or temporally detrended data), but preprocessing is not a part of TDT, as numerous software packages exist that have been created for that purpose, each with their own benefits and drawbacks. For example, detrending data or scaling time-series are important steps for single image decoding, but this can be done with popular software packages including SPM, FSL, or AFNI or directly in Matlab with specialized toolboxes. The same applies to visualization of results: third-party software can be used for that purpose, for example MRIcron (Rorden, 2007) to visualize searchlight accuracy maps or weight maps.

### Comparison to existing packages of multivariate data analysis

A few other toolboxes have been created that can be used to carry out multivariate decoding analyses on fMRI data. The key advantages of TDT have been listed in the introduction. In general, it is difficult to tell to which degree other toolboxes offer similar advantages, for example where they might be comparably transparent, fast, easy to use, or versatile. More objective measures that have been used for comparison purposes have partly underestimated the ability of other toolboxes (Schrouff et al., 2013; Grotegerd et al., 2014) because the toolboxes might be more elaborate than described on the toolbox websites. Rather than thoroughly describing the advantages and disadvantages of all existing toolboxes, we will mention the most widely used and in our view most promising alternative toolboxes and try to elucidate some degree of comparison to TDT.

The Princeton MVPA toolbox (http://code.google.com/p/princeton-mvpa-toolbox/) is a rather versatile tool with a number of classifiers, basic feature selection capabilities and scaling and the possibility to run searchlight analyses, but it requires a more advanced level of programming skills and to our knowledge is not further developed. Another advantage of this toolbox is an active user community. TDT on the other hand offers more analysis methods, can be easily extended and due to the multilevel approach is probably easier for getting started. The SPM interface of TDT is particularly well suited for users of SPM.

The developers of the Princeton MVPA toolbox seem to have shifted their focus to PyMVPA (Hanke et al., 2009a,b), a highly versatile programming environment that allows for a large variety of decoding analyses. The key advantages of this toolbox are the high level of sophistication, the active user community and the fact that the toolbox does not require third-party software. While TDT requires the third-party software Matlab and might not be as versatile in its core functions, PyMVPA also requires more advanced programming skills and for searchlight analyses is much slower than TDT. In addition, Matlab is still widespread in the neuroimaging community, and running decoding analyses on SPM results from Python is not straightforward. Finally, TDT can also be easily extended if users need additional functionality, which also requires only little programming skills (see Example 3: Extend Toolbox to External Machine Learning Package or More Complex Processing Streams).

A more recent development is PRoNTo (Pattern Recognition of Neuroimaging Toolbox, Schrouff et al., 2013) which has the benefit of a graphical user interface and which also comes with an SPM interface. However, the structure of the toolbox is not optimized for searchlight analyses, and parameter selection or feature selection have not been implemented. Although TDT does not offer a graphical user interface, we are currently working on an interface through the SPM batch system which should become available with the next release of the toolbox and which obviates any command line programming knowledge.

We would also like to mention a toolbox specifically created for carrying out representational similarity analysis (RSA toolbox, Nili et al., 2014) which can serve as a standard for this type of analysis. The capabilities of TDT for representational similarity analysis at the current stage are still much more basic than those of the RSA toolbox. However, TDT is in our experience faster than the RSA toolbox, both for searchlight analyses and for creating correlation matrices. Users who would like to benefit from the speed of TDT, but use all functionalities of the RSA toolbox might consider using both toolboxes. Alternatively, appropriate extensions can be made (see How to Extend the Toolbox) to include many functionalities of the RSA toolbox. We are planning to include more representational similarity analysis methods in future versions of TDT.

A very recent development under Matlab is CosmoMVPA (http://cosmomvpa.org/) which is still in its very early stage. The general structure of the toolbox is quite similar to TDT. Rather than creating a *cfg* in the beginning as is the case for TDT, each option is passed along with the multiple subfunctions that are called. The toolbox offers a range of classifiers, allows volumetric and surface-based searchlight analysis and has a basic interface to SPM and other brain analysis software packages. The toolbox is easy to use with some intermediate level programming knowledge in Matlab. However, CosmoMVPA currently does not offer decoding designs, potentially leading to overlooked mistakes in decoding analyses. Due to its novelty, the error management of the toolbox and additional methods such as feature selection and parameter selection are still quite basic. Passing parameters along the use of subfunctions also invites mistakes on the side of the user. However, it is possible that these current limitations will be changed in future versions of the toolbox.

Finally, for completeness we would like to mention additional MVPA software packages for fMRI data: Searchmight (http://www.princeton.edu/~fpereira/searchmight/) which is a dedicated and fast software package for simple searchlight analyses with different classifiers^3^, 3dsvm (http://afni.nimh.nih.gov/pub/dist/doc/program_help/3dsvm.html) which is part of the software package AFNI; PROBID (http://www.kcl.ac.uk/iop/depts/neuroimaging/research/imaginganalysis/Software/PROBID.aspx) which is specialized for group comparisons, MANIA (Grotegerd et al., 2014), and the brain decoder toolbox (http://www.cns.atr.jp/dni/download/brain-decoder-toolbox/).

## Conclusions

TDT offers a user-friendly, yet powerful and flexible framework for the multivariate analysis of brain imaging data. The toolbox has many advantages in terms of structure, transparency, speed and error management. It comes with an interface to the common brain data analysis software SPM which should make it particularly easy to apply to existing data. Beginners can start using the toolbox with one single line of code, which increasingly can be extended to exploit the full functionality of TDT. In addition, the toolbox can easily be extended for more general purpose use which allows adding new classifiers, feature selection methods, or even complete external software packages for multivariate data analysis. We hope that TDT—through its simplicity and flexibility—will encourage a much broader application of machine learning methods in the analysis of functional imaging data.

## License statement

TDT can be downloaded from http://www.bccn-berlin.de/tdt. The toolbox code is open source, but is licensed as copyright software under the terms of the GNU General Public License (Free Software Foundation). In addition, the toolbox comes with third-party software (LIBSVM, LIBLINEAR, Newton-SVM, http://research.cs.wisc.edu/dmi/svm/nsvm/), each with their respective copyright. TDT has been tested under Windows, Linux, and OS X and works both on 32 and 64 bit systems. The toolbox is compatible with Matlab versions R2006b or above and requires no additional Matlab toolboxes. It works “out-of-the-box” with an installed version of SPM2, SPM5, SPM8, or SPM12b. In addition, we provide an example data set that can be downloaded from the toolbox website.

### Conflict of interest statement

The authors declare that the research was conducted in the absence of any commercial or financial relationships that could be construed as a potential conflict of interest.

## Footnotes

^1^To support this observation numerically, we conducted a Google Scholar search where we intended to limit our search to articles using multivariate decoding methods and which explicitly mention the software package they use for regular analyses. These articles should also mention the MVPA toolbox used. For the search, we used the combination “fMRI AND (‘multivariate pattern analysis’ OR ‘multivoxel pattern analysis’) AND (FSL OR AFNI OR Freesurfer OR SPM).” This yielded 659 hits between 2010 and 2013. Of these, only 103 (15.6 %) contained the strings “PRoNTo,” “PyMVPA,” “MVPA toolbox” or “3dsvm,” which refer to previous MVPA-related software packages (see Comparison to Existing Packages of Multivariate Data Analysis). 132 (20.0 %) contained the terms “in-house,” “custom-written,” “custom-made,” “custom-built” or “custom Matlab.” Neither term was contained in 442 (67.0%) articles.

^2^Please note that one-vs.-one multiclass SVM classification usually follows a “winner-takes-all” voting scheme. In case of ties, LIBSVM automatically assigns the first class to the tied output. To circumvent this bias, TDT resolves ties by choosing the class with the maximum decision value over all pairs of comparisons.

^3^It is difficult to compare the Searchmight and TDT in terms of speed. Since the major bottleneck in processing speed is the classifier, this will depend crucially on the implementation. For example, Searchmight comes with very fast and dedicated Gaussian Naïve Bayes searchlight capabilities, but at the same time does not pass kernel matrices to LIBSVM which can slow down processing.
